# Fractures of the Cervix Scapulæ

**Published:** 1845-02

**Authors:** 


*Fractures of the cervix scapulæ.*—Mr. Hancock, in the Provincial Medical Journal, says, while speaking of fractures of the neck of the scapula, that they resemble dislocations into the axilla in general appearance; in the projection of the acromion process; the depression and flattening beneath this process; the loss of power, and elongation of the limb. They differ from dislocation, in the freedom with which the surgeon may move the arm in all directions; the facility of bringing it to the side; the crepitus felt when the parts are restored to their natural situation, and which can best be felt, by placing the finger or thumb upon the coracoid process, whilst the arm is rotated; the facility with which the deformity is overcome by merely pushing the arm upwards and a little outwards; and lastly, the return of the deformity immediately the arm is allowed to hang unsupported. When these signs are present, the case is one of fracture, and not dislocation.—*Lond. Med. Times.*

